# Treatment of Spina Bifidia

**Published:** 1849

**Authors:** Daniel Brainard

**Affiliations:** Professor of Surgery in Rush Medical College


### ARTICLE VIII.

*Treatment of Spina Bifidia by Injection of Tinct. Iodine.* By DANIEL BRAINARD, M.D., Professor of Surgery in Rush Medical College.

In the No. of this Journal for Jan., 1848, a case was reported of treatment of this defect of the spinal column by injections. The case, at the time, I considered cured; the sac being collapsed so that the long opening into the spinal canal could be distinctly felt. This perfect obliteration of the tumor was, however, effected under the use of firm and steady pressure. Immediately after that report was made, the public authorities removed the patient from under my care to the county poor house where the pressure was entirely discontinued. Under this state of things, the tumor re-appeared, and attained, after a time, half its original size, as near as I could learn. I then requested Dr. Huber, the physician of the poor house, to apply the same remedy as before; which he did, with the exception that the regulations of that establishment were not such as to permit of pressure being used. This I did not regret, as of those who had witnessed the former treatment, some have considered the improvement to be due to the pressure alone, while others, admitting the good effect of the injection, consider it, nevertheless, as a dangerous operation. It was, therefore, fortunate that so favorable a case should present, in which the effect of the tr. iodine could be tried alone. That ʒjjj, of a solution of the strength given below or ʒjss of double strength, could be injected repeatedly into the sac of the arachnoid membrane, without producing more than "slight febrile symptoms," will doubtless surprise some whose views of the extreme sensitiveness of serous membranes, are formed from their actions in a state of health.

It is, however, in accordance with all we know of the action of irritants upon serous surfaces upon which dropsical effusions take place, in which the production of the inflammation is difficult in direct proportion to the amount of distention and the length of time it has existed.

The case being now cured, so far as the obliteration of the sac is concerned, it will only remain to endeavor to remedy the imperfect action of the muscular and nervous systems, indicated by the incontinence of the urine and fæces, and the weakness of the lower limbs. For this purpose we have requested Dr. Huber to administer the 20th of a grain of strychnine thrice daily until its specific action is induced. By then discontinuing it and renewing after a few days so as to keep the system under its influence most of the time for many months, there is good reason to hope that a favorable result may be obtained.

*Dear Sir:*—After the case of spina bifidia came into my hands, I continued the treatment which you had begun.

I injected the tumor thirteen times, viz.: May 3, 10, 20; June 15, 22; July 14; August 10, 15, 25; September 5, 16, 26; and October 20, 1848. The injection for the first four times was of the strength of four grains iodine and sixteen grains iodide potassium to ℥j distilled water, beginning with ʒjss and increased to ℨiij at the fourth injection, the sac had then become so much contracted, that the latter quantity could with difficulty be forced in, I therefore doubled the strength of the solution and injected but ℨjss. As the sack decreased the difficulty of forcing in the liquid increased, so that on the last three or four occasions, upon removing the syringe I found it full above the piston, the resistance to the entrance of the; liquid into the tumor having forced it back.

For the first three times I used a small trocar and canula the walls of the tumor had by this time become so thickened by the deposit of lymph on their interior, that the trocar could not conveniently be forced through. I then substituted a darning needle, set into a handle, into the puncture of which I introduced the point of the fistula lachrymalis syringe.

The tumor is now one and a half inches in its smaller, and one and three fourth inches in its larger diameter, projecting about three fourths of an inch; its surface is irregularly nodulated and very firm, seemingly semi-cartilaginous, creaking when pierced by the needle.

After the first two operations, the child had some slight febrile symptoms, but not since. She has improved in the use of her lower extremities, being now able to walk across the room. Her capability of retaining the contents of her rectum and bladder has slightly improved.

So far as regards the tumor, I consider the cure complete. The shrunken and thickened sac confining the serum perfectly in the cavity of the spine, and offers much encouragement in the treatment of these hitherto hopeless cases.

Yours respectfully,

H. S. Huber.

Chicago, Jan. 23, 1849.

P. S. I consider the injection as the cause of the improvement, as I could not get pressure applied so as to be of any service. I think if pressure had been steadily and regularly applied it would have much expedited the cure. H. S. H.

